# Management of medication overuse (MO) and medication overuse headache (MOH) S1 guideline

**DOI:** 10.1186/s42466-022-00200-0

**Published:** 2022-08-29

**Authors:** Hans-Christoph Diener, Peter Kropp, Thomas Dresler, Stefan Evers, Stefanie Förderreuther, Charly Gaul, Dagny Holle-Lee, Arne May, Uwe Niederberger, Sabrina Moll, Christoph Schankin, Christian Lampl

**Affiliations:** 1 Abteilung Für Neuroepidemiologie, Institut Für Medizinische Informatik, Biometrie Und Epidemiologie (IMIBE), Universität Duisburg-Essen, Hufelandstraße 55, 45147 Essen, Germany; 2 Institut Für Medizinische Psychologie Und Medizinische Soziologie, Universitätsmedizin Rostock, Zentrum Für Nervenheilkunde, Gehlsheimer Straße 20, 18147 Rostock, Germany; 3 Klinik Für Psychiatrie Und Psychotherapie, Graduiertenschule & Forschungsnetzwerk LEAD, Universität Tübingen, Calwerstraße 14, 72074 Tübingen, Germany; 4 Chefarzt der Neurologischen Klinik II, Krankenhaus Lindenbrunn, Postfach 1120, 31861 Coppenbrügge, Germany; 5 Neurologische Klinik, Ludwig-Maximilians-Universität München, Neurologischer Konsildienst Der LMU, Innenstadtklinikum, Ziemssenstraße 1, 80336 Munich, Germany; 6 Kopfschmerzzentrum Frankfurt, Dalbergstr. 2a, 65929 Frankfurt, Germany; 7 Klinik Für Neurologie Und Westdeutsches Kopfschmerzzentrum, Universitätsklinikum Essen, Hufelandstraße 55, 45128 Essen, Germany; 8 Institut Für Systemische Neurowissenschaften, Universitätsklinikum Hamburg, Eppendorf (UKE), Gebäude W34, 3. Stock, Martinistraße 52, 20246 Hamburg, Germany; 9 Institut Für Medizinische Psychologie Und Medizinische Soziologie, Universitätsklinikum Schleswig-Holstein, Campus Kiel, Preußerstraße 1- 9, 24105 Kiel, Germany; 10 Psychologische Praxis, Rothschildallee 16, 60389 Frankfurt am Main, Germany; 11 Oberarzt, Universitätsklinik Für Neurologie, Inselspital Bern, Freiburgstrasse 4, 3010 Bern, Germany; 12 Ordensklinikum Linz, Krankenhaus der Barmherzigen Schwestern Linz Betriebsgesellschaft M.B.H., Seilerstätte 4, 4010 Linz, Germany

**Keywords:** Migraine, Migraine attack, Medication overuse, Medication overuse headache, Management

## Abstract

**Introduction:**

Chronic headache due to the overuse of medication for the treatment of migraine attacks has a prevalence of 0.5–2.0%. This guideline provides guidance for the management of medication overuse (MO) and medication overuse headache (MOH).

**Recommendations:**

Treatment of headache due to overuse of analgesics or specific migraine medications involves several stages. Patients with medication overuse (MO) or medication overuse headache (MOH) should be educated about the relationship between frequent use of symptomatic headache medication and the transition from episodic to chronic migraine (chronification), with the aim of reducing and limiting the use of acute medication. In a second step, migraine prophylaxis should be initiated in patients with migraine and overuse of analgesics or specific migraine drugs. Topiramate, onabotulinumtoxinA and the monoclonal antibodies against CGRP or the CGRP-receptor are effective in patients with chronic migraine and medication overuse. In patients with tension-type headache, prophylaxis is performed with amitriptyline. Drug prophylaxis should be supplemented by non-drug interventions. For patients in whom education and prophylactic medication are not effective, pausing acute medication is recommended. This treatment can be performed in an outpatient, day hospital or inpatient setting. Patients with headache due to overuse of opioids should undergo inpatient withdrawal. The success rate of the stepped treatment approach is 50–70% after 6 to 12 months. A high relapse rate is observed in patients with opioid overuse. Tricyclic antidepressants, neuroleptics (antiemetics) and the administration of steroids are recommended for the treatment of withdrawal symptoms or headaches during the medication pause. Consistent patient education and further close monitoring reduce the risk of relapse.

## What is new?

- The International Headache Society (IHS) classification of headache specifies medications that can cause Medication Overuse Headache (MOH).
- The global prevalence of MOH is between 0.7 and 1%.
- The societal costs of treating MOH are three times higher than those of treating episodic migraine.
- The most important risk factors for MOH are: pre-existing primary headache, e.g. migraine or tension-type headache, female gender, > 10 headache days per month, low social status, other chronic pain disorders, stress, physical inactivity, obesity, smoking, dependent behaviour and other psychiatric disorders such as depression or anxiety disorder.
- The monoclonal antibodies against CGRP or the CGRP-receptor, topiramate and onabotulinumtoxin A, are effective in the prophylaxis of chronic migraine. This is also true for patients with headache due to medication overuse.
- Non-drug measures complement drug prophylaxis in MOH.
- The greatest risk of relapse is in the first year after a medication pause or withdrawal.

## The most important recommendations at a glance

**Fig. 1:**
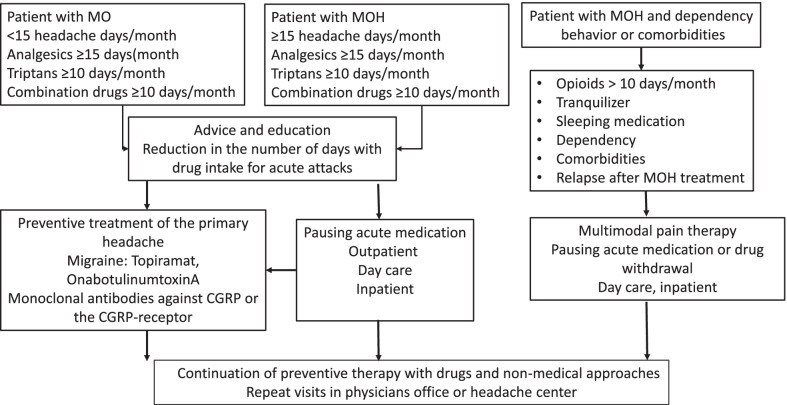
Step care approach to the treatment of MO and MOH

Treatment of headache due to overuse of analgesics or specific migraine medications involves several stages (Fig. 1):

- Patients with medication overuse (MO) or medication overuse headache (MOH) should be educated about the relationship between frequent use of symptomatic headache medication and chronicity of headache, with the goal of reducing and limiting the use of acute medication.
- As a second step, prophylaxis should be initiated in patients with migraine and MOH. Topiramate, onabotulinumtoxin A, and the monoclonal antibodies against CGRP or the CGRP-receptor are effective during existing medication overuse.
- In patients with tension-type headache drug prophylaxis with amitriptyline is recommended.
- Drug prophylaxis should be supplemented by non-drug methods.
- In patients for whom education and drug prophylaxis are not sufficient, medication pause performed in an outpatient, day-case or inpatient setting, depending on the constellation is recommended.
- Patients with headache due to overuse of opioids should undergo inpatient withdrawal treatment.
- The success rate of stepped therapy is about 50–70% after 6–12 months. There is a high relapse rate, especially in patients with opioid overuse.
- Tricyclic antidepressants, neuroleptics (antiemetics), and administration of steroids are recommended to treat withdrawal symptoms or headaches during the medication break.
- Consistent patient education and further close monitoring reduce the risk of relapse.

## Introduction

### Need for the guideline

Medication overuse headache (MOH) has a prevalence of 0.7–1% in Germany [1]. The cost of treatment is three times higher than that of episodic migraine [2]. Guidelines for diagnosis and treatment are of high practical relevance for patient care.

### Aims of the guideline

To provide evidence-based recommendations for the prevention and treatment of headache associated with the overuse of analgesics or specific migraine medications.

### Target audience

Headache specialists, neurologists, pain therapists, pain psychotherapists, general practitioners, pharmacists.

### Areas of care

The guideline is applicable in outpatient, day-care and inpatient settings.

### Key words

Medication overuse (MO), medication overuse headache (MOH), chronic migraine, chronic tension-type headache, prophylaxis, prevention, therapy.

Frequent or daily use of medications to treat acute headaches can lead to an increase in headache frequency and a transition from episodic to chronic headaches. Medication overuse (MO) describes the overuse of medications to treat acute headache. Medication overuse headache (MOH) is defined as headache occurring ≥ 15 days per month and associated with the use of specific headache medications (triptans, ergot alkaloids), mixed analgesics on 10 or more days per month, or non-opioid analgesics (such as NSAIDs or acetylsalicylic acid, acetaminophen) on 15 or more days per month [3]. This joint guideline from the German Society of Neurology (DGN) and the German Migraine and Headache Society (DMKG) answers the following questions:

- How can MOH be prevented?
- Is education effective in the management MOH?
- Are pharmacological treatment and/or nonmedication prophylaxis effective the treatment of MOH?
- Is a medication pause or withdrawal effective for the treatment of MOH?
- How can symptoms be managed during medication pause or withdrawal?
- How can relapse be prevented after treatment of MOH?

## Definitions and nomenclature

Chronic headache due to overuse of analgesics or specific migraine medications is defined by the International Headache Society (IHS) criteria as headache that persists on 15 or more days per month for a period of at least 3 months and is precipitated by regular use of symptomatic headache medication on at least 10 or 15 days per month [3]. In this context, the IHS defines this headache as a secondary headache. However, MOH can also be considered a complication of an underlying primary headache because overuse usually leads to a transition from episodic to chronic headache or changes its clinical presentation [3]. The underlying primary headache (usually migraine) and the headache from overuse of analgesics or migraine medications should result in two separate diagnoses.

The diagnostic criteria include:

A) headache on ≥ 15 days/month in a patient with a pre-existing headache disorder.
B) Regular overuse for more than 3 months of one or more medications taken for acute or symptomatic treatment of headache.

Overuse is defined as:

C) Intake of non-opioid analgesics (such as NSAIDs or acetylsalicylic acid, acetaminophen) on ≥ 15 days/month; or
D) Intake of combination analgesics (taking substances from different classes), triptans, ergot alkaloids, or opioids on ≥ 10 days/month.

The International Headache Classification ICHD-3 prompts identification of overused substances at diagnosis [3] and differentiates between simple analgesics, combination analgesics [4], ergot alkaloids [5], triptans [6] and opioids [7]. Patients who take specific migraine medications or combination analgesics on 10 or more days per month but have headaches on fewer than 15 days per month are diagnosed as medication overuse (MO) [3].

A causal relationship between increasingly frequent or daily use of acute medications and chronic medication overuse headache can only be demonstrated if the frequency of headaches decreases with reduced use of acute medications. However, unlike previous versions, ICHD-3 no longer requires improvement in headache with reduction in days of acute medication use as a prerequisite for the diagnosis of MOH. However, decreasing efficacy of acute medication in MO is often observed in the early phases of headache with medication overuse.

Frequent use of acute medications does not lead to chronic headache in all cases. There are patients who take triptans 10 or more days a month for many years without developing chronic migraine and without an increase in headache frequency. This constellation is called overuse of analgesics or migraine medications (MO).

## Epidemiology

The prevalence of MOH is approximately 0.5–2.0% of the population [8, 9]. Adolescents may also be affected [10]. The definition of headache due to the overuse of analgesics or migraine medications has changed several times over the years. Therefore the prevalence depends on the definition used and the numbers are fluctuating [11]. The prevalence of MOH in Germany is between 0.7 and 1% [1, 12, 13]. In Germany, about 40–50% of all patients with chronic headache overuse analgesics or specific migraine medications, and about the same proportion of all patients treated annually with chronic headache in headache centres have MOH. Prospective studies in specialized headache centres showed that between 3 and 14% of all patients with primary episodic headache develop chronic headache within 1 year [14].

## Diagnosis and clinical criteria

The headache characteristics of MOH depend on the underlying primary headache [15]. Migraineurs who take triptans too frequently usually report a migraine-like daily headache or an increase in migraine frequency. In some patients, the phenotype of migraine changes with increasing attack frequency. The autonomic symptoms associated with migraine decrease in intensity. Patients with chronic headache who overuse analgesics report an increase in the number of days with tension-type headache symptoms [15].

The speed with which MOH develops also depends on the substance taken. MOH develops significantly faster when triptans, opioids and combination analgesics are taken compared with simple analgesics [15]. This observation was confirmed by a French study of 82 patients who used triptans too frequently [16]. In a population-based study in the United States in 24,000 headache patients, opioids and barbiturates in particular increased the risk of chronic daily headache [17]. A systematic literature review of 29 studies confirmed a particularly high risk of MOH with opioid use [18]. The risk of developing MOH with overuse of combination drugs containing caffeine is difficult to assess compared to analgesics without adjuvant caffeine, as the consumption of caffeine is high in the population [19].

The diagnosis of MOH is based on history, diagnosis of pre-existing primary headache disorder and days of headache medication use documented in a headache diary. Most patients with MOH initially had migraine or, less commonly, tension-type headache [7, 20]. Patients with cluster headache usually develop do not develop MOH even with multiple daily use of triptans. However, a small number of patients with cluster headache and MOH have been described in the literature. They also had migraine or a positive family history of migraine [21, 22]. For patients with chronic headache and medication overuse without pre-existing episodic headache, with focal neurological symptoms or neuropsychological abnormalities, or onset of chronic headache beyond age of 50 years other secondary headache disorders must be ruled out by appropriate imaging or laboratory tests.

## Prevention of MOH

**Table:** 

Recommendations Patients at increased risk for developing MOH can be identified through GPs, pharmacists or evaluation of prescriptions. In these patients, it is important to monitor prescription and over-the-counter medications and refer them to a headache specialist in a timely manner. Risk factors for developing MOH should be considered. Training of staff in doctors' offices (headache nurses) and pharmacies can help improve care	

A number of epidemiological studies investigated risk factors for the development of MOH. These risk factors include primary headaches, such as migraine and tension headache, female gender, history of > 10 headache days per month, low social status, other chronic pain disorders, stress, physical inactivity, obesity, smoking, overuse of substances and psychiatric conditions such as depression or anxiety disorders [23, 24]. In a population-based study in Norway with 5183 participants and an observation period of 11 years, the incidence of a MOH was 0.72 per 1000 person-years. In a multivariate analysis, the risk of developing an MOH was increased in patients who regularly took benzodiazepins, or who suffered from chronic musculoskeletal pain, depression or anxiety disorders [25]. Smoking and physical inactivity doubled the risk [25]. A German multicenter study showed that an education programme with minimal contact including cognitive-behavioural methods either in a group setting or with written instructions can reduce the risk of developing an MOH in patients at risk [26].

## Education and counselling to treat MOH

**Table:** 

Recommendations: • In a proportion of patients with MOH, regardless of age, counselling and education are sufficient to treat MOH. This applies for patients who take triptans or simple analgesics as monotherapy and who do not suffer from severe psychiatric comorbidity • Education can be implemented by general practitioners, anesthetists, neurologists, pain therapists, pain psychotherapists, pharmacists and headache nurses • After successful withdrawal therapy, the indication for migraine or tension headache prophylaxis needs to be reassessed • If overuse does not stop, further treatment steps, including migraine prophylaxis, must be initiated • Education and training are usually not effective in patients who are overusing opioids or who have relapsed after previous withdrawal treatment. These patients should receive multimodal care in a headache centre or as inpatients, with additional psychological counselling	

This section presents studies of treatment options whose main component is the provision of information to patients, described by the terms education, counselling, psychoeducation and training. An Italian study from 2006 compared the effectiveness of counselling as monotherapy with an outpatient or inpatient drug withdrawal programme. The study included 120 patients with MOH and migraine as the primary headache disorder [27]. Education was as effective as outpatient or inpatient medication withdrawal, with a success rate of over 70% at 2 months. A second study from 2013 in 137 patients with MOH and migraine as the primary headache disorder compared the effectiveness of an education programme with two structured pharmacological withdrawal programmes [28]. The success rate in reducing medication days to less than 15 days per month was 61% in the first two treatment groups and 89% in the third group. The difference with the first two groups was statistically significant. Similar results were found for headache parameters, which improved most significantly in the third group [28]. Similar results were found for headache parameters, which improved most significantly in the third group.

In a prospective, 18-month study in Norway, 109 patients with MOH and chronic headache, mostly tension-type headache, received written information about the role of overuse of analgesics or migraine medications in headache chronification [29]. At the end of the observation period, headache days had decreased from 22 to 6 per month. 76% of patients no longer overused medication. Another study implemented this treatment programme in general practitioners' practices [30]. The group of patients with counselling reduced their headache days on average from 25 to 17 per month and also reduced those days on which they used acute medications from 24 to 13 per month. The control group without counselling (treatment as usual) showed no changes. The authors concluded that brief intervention in primary care was effective and had the potential to save resources that would be needed for treatment-resistant cases in neurological care. The only relevant predictors of worse outcomes were initially increased headache frequency and medication adherence [31].

A study in Italy showed that of the patients with MOH who received education about clinical features and risk factors of MOH 4 weeks before randomisation to a withdrawal trial, approximately 25% no longer met the criteria for MOH at the time of randomisation. These patients thus treated themselves [32]. In another study, Krause et al. investigated the effectiveness of a 3-week outpatient interdisciplinary programme in which patients were counselled by neurologists and psychologists [33]. The study enrolled 379 patients. One-year follow-up data were available for 152 patients. Headache intensity on a numerical rating scale (NRS) of 1 to 10 averaged NRS 6.1 at baseline, NRS 3.5 at discharge and NRS 3.3 at 1 year. The score measuring functional limitation due to migraine improved significantly over 1 year. Depression and anxiety scores also improved significantly over 1 year [33].

In a 6-month study involving 100 headache patients, the effectiveness of didactic instruction on migraine pathogenesis and related disease management was investigated [34]. The results were clearly in favor of the group that participated in the education. In another study, 26 children and adolescents with chronic headache were examined over a 3-year period in relation to daily use of pain medication [35]. Weekly medication intake averaged 28 tablets (range 19–41). All children and parents were informed about the concept of MOH and encouraged to take a medication pause through psychoeducational information. Successful withdrawal was achieved in 25 of 26 patients without hospitalization or significant impairment of quality of life and with improvement of previously chronic daily headaches.

A study in France investigated the interaction of MOH and individual predispositions to dependence [36]. Functional imaging, biological and pharmacogenetic studies suggest that the pathophysiological mechanisms of MOH overlap with those of substance-related disorders. Based on these data, MOH patients are divided into two subgroups: one group in which overuse was mainly due to exacerbation of the headache course, and another group in which psychosocial behavioural problems were a major determinant of overuse. A study by Wallasch et al. in 204 headache patients including 68 with an MOH showed that the combination of medication pause and psychological treatment had the greatest positive effect on headache frequency [37].

Increasing knowledge about MOH in the general population may also be a goal of public education. A large-scale 4-month campaign in Denmark involving relevant stakeholders (e.g. pharmacist associations, patient associations) showed that it was possible to increase the proportion of the population aware of MOH from 31 to 38%. Social media seems to play a special role here [38].

In summary, several studies showed that education and training of patients with overuse of analgesics or migraine medications are effective therapies (Table 1).

**Table 1:** Success rates in studies of education or training as therapy or part of a therapeutic approach (MOH = medication overuse headache)

N	Diagnosis	Follow-up (months)	Success rate (%)	References
120	Migraine/MOH	2	70	[27]
137	MOH	2	81	[28]
109	MOH	18	76	[29]
60	MOH	6	92	[39]
100	Migraine/MOH	6	64	[34]

## Drug and/or non-drug prophylaxis for the treatment of MOH

**Table:** 

Recommendations • Patients at risk for MOH and/or for whom education and training are not sufficiently effective should receive prophylaxis with drugs for the underlying headache disorder • For migraine, evidence for efficacy of prophylaxis despite concomitant MOH has been shown for topiramate, onabotulinumtoxinA, and the CGRP and CGRP-receptor antibodies • Drug prophylaxis of migraine should be supplemented by additional nonpharmacologic therapy. Multimodal approaches are most effective. However, there are only few randomized trials that have compared the combination of drug and non-drug therapy in patients with MO and MOH • Migraine patients with MOH in whom drug prophylaxis with topiramate or onabotulinumtoxinA is not effective, not tolerated, or contraindicated should be treated with a monoclonal antibody against CGRP or the CGRP-receptor. In this case, current reimbursement guidelines must be followed, regardless of approval	

Efficacy for drug treatment despite persistent MO and MOH has been demonstrated for topiramate, onabotulinumtoxinA, erenumab, galcanezumab, fremanezumab, and eptinezumab.

The efficacy of topiramate has been evaluated in clinical trials in Europe and the United States in patients with chronic migraine [40–42]. In the European trial, patients received topiramate at doses up to 200 mg/d without prior stop of acute medication overuse. The MOH subgroup showed a significant reduction in the mean number of migraine days, compared with placebo. The number of days with acute medication use was also reduced in the topiramate group, although the difference from the placebo group did not reach statistical significance [40]. Post-hoc analysis of the USA study showed a nonsignificant reduction in mean monthly migraine days in the MOH subgroup, compared with placebo [41, 42]. Another study showed a significant reduction in headache days and acute medication use days in the topiramate group, compared with placebo [43]. A major limitation of all studies on topiramate is the high drop-out rate in the topiramate groups due to side effects.

OnabotulinumtoxinA has been evaluated in two large randomized, placebo-controlled trials for efficacy in the prophylactic treatment of chronic migraine [44, 45]. In these trials, approximately 65% of patients met criteria for MOH. Patients with opioid overuse were excluded from the studies. After 24 weeks, there was a statistically significant reduction of 8.2 headache days with onabotulinumtoxinA, compared with 6.2 days with placebo. Significant differences were also found for the frequency of migraine days, days with moderate and severe headache, and cumulative headache hours on days with headache. Patients with MOH did not respond in a similar way to treatment with onabotulinumtoxinA than those with chronic migraine without MOH [46].

The monoclonal antibodies against CGRP (eptinezumab, fremanezumab, galcanezumab) and against the CGRP-receptor (erenumab) have been evaluated for prophylactic efficacy in patients with chronic, and in some cases episodic, migraine with and without MOH or medication overuse (MO) in large randomized placebo-controlled trials (Table 2).

**Table 2:** Efficacy of monoclonal antibodies against CGRP or the CGRP receptor in the therapy of MOH

Drug	Dose	Reduction of migraine days/month	50%-responder rate for migraine days (%)
Fremanezumab	1 × Quarterly	− 4.7	35
Fremanezumab	1 × Monthly	− 5.2	39
Placebo		− 2.5	14
Erenumab	70 mg	− 6.6	36
Erenumab	140 mg	− 6.6	35
Placebo		− 3.5	18
Galcanezumab	120 mg	− 4.8	28
Galcanezumab	240 mg	− 4.5	28
Placebo		− 2.2	15
Eptinezumab	100 mg	− 8.4	60
Eptinezumab	300 mg	− 8.6	62
Placebo		− 3.0	14

Erenumab significantly reduced the number of migraine days in patients with chronic migraine and medication overuse in a subgroup analysis [47]. A total of 667 patients were studied, of whom 41% (n = 274) met criteria for MO. Patients were treated with 70 mg or 140 mg erenumab or placebo. In the MO subgroup, both erenumab groups (70, 140 mg) significantly reduced the mean number of monthly migraine days at month 3 (− 6.6; 95% CI − 8.0 to − 5.3 and − 6.6; 95% CI − 8.0 to − 5.3), compared with placebo (− 3.5; 95% CI − 4.6 to − 2.4), and the number of days with migraine-specific acute medication, − 5.4 days; 95% CI − 6.5 to − 4.4 and − 4.9; 95% CI − 6.0 to − 3.8 vs. − 2.1; 95% CI − 3.0 to − 1.2. Treatment with erenumab achieved a ≥ 50% reduction in migraine days in 35% and 36% of patients at doses of 70 mg and 140 mg, respectively, compared with only 18% in the placebo group.

A post-hoc subgroup analysis of the EVOLVE-1 and EVOLVE-2 (pooled) trials and the REGAIN phase III trial evaluated the efficacy of galcanezumab in patients with episodic migraine and chronic migraine with MO [48]. The use of opioid- and barbiturate-containing medications was allowed but limited to 3 days per month during the studies. At baseline, the proportion of patients with MO in the placebo, galcanezumab 120-mg, and 240-mg groups was 19.4%, 17.3%, and 19.3%, respectively, for EVOLVE-1/-2 (pooled; post hoc) and 63.4%, 64.3%, and 64.1%, respectively, for REGAIN (a priori). Both the 120-mg and 240-mg galcanezumab doses significantly reduced mean monthly migraine days compared with placebo in patients with MO (p ≤ 0.001). In addition, both galcanezumab doses reduced the proportion of patients with MO (p ≤ 0.001).

The CONQUER study, which demonstrated the efficacy of galcanezumab as migraine prevention in patients who had previously failed up to 4 prophylactic treatments, also demonstrated a clinically relevant reduction in days of use of acute headache medications. The greatest reduction was observed for triptans, followed by NSAIDs and acetylsalicylic acid [49].

Fremanezumab was evaluated in the 12-week phase III HALO study in patients with chronic migraine and MO. This involved treatment with fremanezumab in two different dose regimens over 3 months: 675 mg/placebo/placebo) or 675 mg/225 mg/225 mg, compared with placebo [50]. Of 1130 patients enrolled, 587 (51.9%) had MO at baseline. Fremanezumab reduced the placebo-adjusted least-square mean monthly headache days by 2.2 (95% CI 3.1–1.2) and 2.7 days (95% CI 3.7–1.8, P < 0.0001) in patients with MO and without MO, respectively. For single quarterly administration, the results were as follows: 1.4 (95% CI 2.3–0.5, P = 0.0026); with monthly administration, 1.4 (95% CI 2.3–0.6, P = 0.0017). Significantly more patients treated with fremanezumab had a 50% reduction in headache days compared with placebo, regardless of whether MO was present at baseline (quarterly: 70/201 (34.8%), monthly: 78/198 (39.4%), placebo 26/188 (13.8%); without MO: quarterly: 71/174 (40.8%), monthly: 75/177 (42.4%) vs. placebo 41/183 (22.4%)). Significantly more patients treated with fremanezumab did not develop MO again (quarterly dose 111/201 (55.2%), monthly dose 120/198 (60.6%)) vs. placebo (87/188 (46.3%)). In patients who no longer had MO after 6 months, this persisted over 12 months of treatment [50].

The FOCUS trial evaluated the efficacy of fremanezumab in a 12-week, randomized, double-blind, placebo-controlled, parallel-group phase IIIb study in adults with episodic or chronic migraine who had been shown to respond inadequately to two to four pharmacologic classes of migraine prophylaxis medications [51]. Results of the subgroup analysis of patients with MO showed that quarterly and monthly administration of fremanezumab resulted in early, sustained, and clinically meaningful reductions in migraine and headache days compared with placebo.

The efficacy, tolerability, and safety of intravenously administered eptinezumab have been demonstrated in two pivotal phase III trials [52, 53]. A subgroup analysis of the PROMISE-2 trial included data from a total of 431 patients who had concomitant chronic migraine and MOH [54]. The use of opioids and barbiturates was limited in the study. Patients received i.v. eptinezumab 100 mg, 300 mg or placebo. During weeks 1–12, there was a greater reduction in monthly migraine days in patients treated with eptinezumab than in the placebo group (with 100 mg: − 8.4 days, with 300 mg: − 8.6 days, with placebo − 3.2 days). The ≥ 50% responder rate with respect to migraine days was 60.4% for the 100 mg eptinezumab dose, 61.9% for 300 mg, and 34.5% for placebo. Total monthly acute migraine attack medication use decreased from 20.6 days/month at baseline to 10.6 days/month during 24 weeks of treatment (49% decrease) for eptinezumab 100 mg, from 20.7 to 10.5 days/month (49% decrease) for eptinezumab 300 mg, and from 19.8 to 14.0 days/month (29% decrease) for placebo [55].

In all studies, the tolerability of the CGRP (receptor) monoclonal antibodies was very good. There are many diseases in which CGRP plays an important role [56]. Safety data for the use of monoclonal antibodies in these patient groups are not yet available. Therefore, in the following conditions, the use of monoclonal antibodies against CGRP or the CGRP-receptor should be considered only on a case-by-case basis after detailed consideration of potential risks and the potential benefits: Pregnancy and lactation, subarachnoid hemorrhage [57], familial aneurysms, inflammatory bowel disease [58], gastrointestinal ulcers, stroke [57], TIA, coronary artery disease, poorly controlled hypertension [59], Raynaud's disease [60–62], COPD, pulmonary hypertension, wound healing disorders [63], and psoriasis [64].

Smaller studies that investigated the efficacy of valproic acid [65], cannabinoids [66], Pregabalin [67], acupuncture [68], and stimulation of the greater occipital nerve [69] in MOH. Due to the methodological weaknesses of these studies, the results are not conclusive and these therapies cannot be recommended. Beta-blockers, flunarizine, and amitriptyline are first-line prophylaxis for high-frequency episodic migraine. They have not been studied in MOH.

In addition to medication, non-pharmacological treatments play an important role in the treatment of MO and MOH including counseling and education, relaxation techniques, aerobic exercise, cognitive behavioral therapy, and biofeedback [70]. For patients with comorbidities or relapse after initially successful medication withdrawal, multimodal approaches involving physicians, psychologists, and physical therapists should be used in an individual or group setting over several sessions. In a study in patients with chronic headache, the prevalence of patients with MOH decreased from 33.8 to 1.6% at 1 year [37]. In a small randomized trial, electromyographic (EMG) biofeedback treatment in combination with drug prophylaxis was compared with drug prophylaxis alone: In the biofeedback group, there were significantly more patients who switched from chronic to episodic migraine. Headache frequency and analgesic use were also reduced, while active coping, measured as functional cognition increased [71]. After a medication pause, mindfulness training was not superior to drug prophylaxis [72]. Nonmedication treatments are particularly appropriate when psychological factors play a significant role in the pathogenesis of MOH. Patients who continued to overuse medication or did not benefit despite cessation of overuse had elevated scores in certain psychopathology or personality scales [73]. In addition, strong correlations emerged between stress and unhealthy lifestyle with MOH [74].

## Pausing medication as treatment of MOH

**Table:** 

Recommendations • Medication pause, drug withdrawal and controlled reduction of acute medication, together with good education, are effective therapies in the treatment of MOH. Their effectiveness is equivalent to that of prophylactic medication • Combination with prophylactic drug therapy for the primary headache disorder is recommended, although studies have not shown superiority over medication pause or withdrawal and drug prophylaxis alone • Patients with MOH for whom prophylactic drug therapy is not effective, not desired or not tolerated should at least take a medication pause or be withdrawn • In the months thereafter, a headache diary should be kept to decide whether prophylactic drug therapy is necessary • The medication pause can begin abruptly in patients taking analgesics or triptans • In patients with overuse of opioids or tranquillizers, medication should be slowly tapered off • In MOH without relevant comorbidity, outpatient withdrawal is possible • In patients with MOH with concomitant diseases, such as depression, anxiety, severe internal disease, abuse of other substances and previous unsuccessful withdrawal from medication, inpatient withdrawal is recommended	

Whether pausing or withdrawing medication break is mandatory for the treatment of MOH is controversial. Currently, there are two therapeutic approaches for MOH and underlying migraine:

1. Medication pause or withdrawal with simultaneous initiation of migraine prophylaxis.
2. Initiation of migraine prophylaxis with topiramate, onabotulinumtoxinA or a monoclonal antibody. If this therapy is effective, medication is discontinued.

There are insufficient data from controlled trials on an appropriate approach for primary tension headache with MOH. An open-label, uncontrolled trial in Denmark tested the efficacy of a 2-month medication break in 337 patients with MOH [75]. Only 2/3 of the patients completed the study. Of these, 45% reported an improvement in headache frequency. Patients with migraine or triptan overuse had better treatment success than patients with tension-type headache [75].

The Norwegian Akerhus study (BIMOH) investigated the effect of a brief intervention by a GP trained in MOH [76]. Patients with MOH were advised to reduce medication and were informed about a possible temporary increase in headache during the reduction phase. Compared with patients who received no intervention, there was a significant reduction in headache and medication frequency in the treatment group. Thus, the recommendation of medication reduction and the education by general practitioners are already effective without the necessity of withdrawal. This effect can still be demonstrated after 16 months [39]. A similar effect was shown in a sub-analysis of the SAMOHA study (Sodium Valproate in the treatment of Medication Overuse HeadAche). Of 122 patients with MOH who were to be included in this study, only 88 patients were randomized after 4 weeks of a prospective baseline phase, as 34 no longer met the criteria for MOH [32].

The question whether drug withdrawal should be in an inpatient or outpatient setting was investigated in an Italian study in patients with chronic migraine and uncomplicated MOH [27]. In this study, outpatient and inpatient medication pauses were equivalent in terms of remission from chronic to episodic migraine and cessation of MOH. However, in complicated MOH, for example when concomitant conditions such as depression, anxiety, further substance abuse and previous unsuccessful medication pauses were present, inpatient withdrawal was superior to outpatient withdrawal or the recommendation of medication reduction [28]. With regard to the long-term outcome 2 years after inpatient withdrawal, no differences were found compared to outpatient withdrawal [77].

The COMOESTAS study recruited 376 patients with MOH in a prospective epidemiological treatment study [78]. Patients were treated with medication pause and prophylactic medication. After 6 months, 2/3 of the patients no longer met the criteria for MOH. In 47% of the patients, chronic headaches had regressed to episodic headaches. When outpatients were compared with inpatients, efficacy was similar, although the discontinuation rate was higher for patients in the outpatient setting [78].

A systematic literature review identified 27 studies investigating the therapeutic success of a medication pause or withdrawal. Nineteen studies started medication prophylaxis in addition to medication pause [79]. Withdrawal was performed either as outpatient, day hospital or inpatient. Initiating migraine prophylaxis with medication in addition to medication pause led to a better long-term outcome than medication pause alone. An open-label study compared three groups: no therapy vs. withdrawal with prophylactic therapy vs. withdrawal without prophylactic therapy [80]. The primary endpoint, change in the number of headache days per month, did not differ between the three types of therapy after 5 months of observation. However, patients who were withdrawn and received prophylactic medication reported the highest benefit from therapy. After 12 months, 53% of patients who received additional prophylaxis showed a ≥ 50% reduction in headache days per month, compared with 25% of patients who received withdrawal alone.

This contrasts with a Danish study comparing 51 patients who were recommended to reduce medication and start prophylaxis with 47 patients who were given structured withdrawal without prophylaxis [81]. Both procedures were effective with 80% of patients without persistent MOH and a 50% reduction in headache frequency. While 85% of the first group continued the prophylaxis they had started, only 62% of the patients in the withdrawal group needed prophylaxis. Therefore, initial withdrawal may obviate the need to start prophylaxis in a subgroup of MOH patients. Finally, another study compared withdrawal plus concurrent initiation of prophylaxis vs. prophylaxis alone without withdrawal vs. withdrawal plus later initiation of prophylaxis after 2 months [82]. The study randomized 102 patients with chronic migraine and MOH. The primary endpoint, change in monthly headache days at 6 months, did not differ between the groups. All three approaches were effective. For secondary endpoints, namely recovery from episodic migraine or "cure" of MOH, the group with withdrawal and concomitant initiation of prophylaxis performed best [82].

The type of withdrawal was prospectively studied in 72 patients with MOH who were randomized either to an abrupt withdrawal group or to a group restricted to 2 days of intake per week. A total of 59 patients were withdrawn. In both groups, there was a significant reduction in headache or migraine days after 6 and after 12 months. The reduction in the abrupt withdrawal group was nominally more significant than that in the restrictive group, without being statistically significant. The study showed an advantage for the abrupt withdrawal group in a secondary endpoint, remission to episodic headache.

The approach to drug withdrawal was prospectively studied in 72 patients with MOH who were randomized either to an abrupt withdrawal group or to a group restricted to 2 days of intake per week [83]. A total of 59 patients were withdrawn. In both groups, there was a significant reduction in headache or migraine days after 6 and after 12 months. The study showed an advantage for the abrupt withdrawal group in a secondary endpoint, remission to episodic headache. In conclusion the abrupt withdrawal seemed to be more effective than a strategy with restriction to two analgesic days per week, although confirmatory studies are still needed.

In summary, inpatient a medication pause is recommended in patients with MOH who overuse opioids or who suffer from psychiatric comorbidity requiring treatment [84, 85]. In these patients, this reflects drug withdrawal, as there is usually dependence and psychological and physical withdrawal symptoms may occur. Abrupt discontinuation of medication is usually not possible, and drug treatment of autonomic withdrawal symptoms is often necessary. In the inpatient setting, not only prophylaxis can be started, but also non-pharmacological therapy strategies and methods of behavioural medicine can be applied.

## Treating symptoms during medication pause or withdrawal?

**Table:** 

Recommendations Tricyclic antidepressants, neuroleptics (antiemetics), and steroids are recommended for the treatment of withdrawal symptoms or headache during the medication pause. This recommendation is based on expert consensus, not controlled trials	

When abruptly discontinuing migraine medications or analgesics, most patients develop a withdrawal syndrome with transient worsening of headache, anxiety and sleep disturbances [7]. Symptoms persist for 2–7 days, depending on the acute medication previously taken [86]. The shortest withdrawal period was observed in patients taking triptans and the longest in patients taking ergot alkaloids or opioids [15]. A number of therapies have been proposed and studied in small observational trials to treat withdrawal symptoms. These therapies included fluid replacement, corticosteroids, neuroleptics, tranquilizers, antiemetics, and simple analgesics reported in a systematic literature review [79]. Three placebo-controlled trials examined the benefit of corticosteroids for the treatment of withdrawal symptoms compared with placebo. One trial was conducted in Norway with 20 to 60 mg of oral prednisolone [87], one in Germany with 100 mg prednisone orally [88] and one in Italy with intravenous administration of 500 mg methylprednisolone [89]. All three studies found no clear therapeutic benefit of prednisone or prednisolone compared with placebo. Another study found no difference between 75 mg prednisone orally and 400 mg celecoxib [90]. Only one large open case series described clinical efficacy of oral prednisone [91]. In a retrospective open study, the combination of intravenous prednisone and diazepam was superior to no therapy [92].

## Preventing relapse after treatment of MOH

**Table:** 

Recommendations • Intensive counseling with motivational interviewing generally assists patients to reduce overuse of headache medication • Patients at high risk of relapse after withdrawal treatments should be identified regarding their risk profile • Regular follow-up is necessary for these patients to prevent relapse. This follow-up is appropriately provided in the form of motivational interviewing • The highest probability of relapse is observed in the first year after drug withdrawal. In this time period Intensive patient care is necessary	

It is important to assess the probabilities of success of the treatment of MO and MOH and avoiding relapse. Several studies and reviews have addressed this issue. In a 2016 systematic literature review, Chiang et al. examined long-term success rates and relapse rates from 22 medication cessation studies with observation periods ranging from 2 to 60 months, with a mean of 12 months. Relapse rates varied from 0 to 45% [79]. Most studies reported relapse rates between 25 and 35%. Predictors of relapse were (a) chronic tension-type headache versus migraine, (b) overuse of triptans, (c) comorbid mental illness, and (d) low socioeconomic status [93]. An Italian study in 188 MOH patients showed, that those with frequent relapse (2 × or more within 3 years, 31% of the sample) were more often hospitalized, more often lived alone, and had lower education level. They also showed higher scores in impairment and depression, lower scores in quality of life, and reported more frequent and severe headaches [93].

The COMOESTAS project demonstrated that of 492 MOH patients assessed 6 months after medication withdrawal, regardless of headache status, 407 no longer overused medication, 23 relapsed, and 62 continued to overuse [94]. A positive predictor was low depression score. Relapse was predicted by prolonged chronic headache. Whether a person could be considered a responder (< 15 headache days/month or > 50% reduction in headache days), was positively predicted by the diagnosis of migraine (compared to tension headache and migraine plus tension headache) and prophylaxis with flunarizine. Individuals who responded particularly well (75% reduction in headache days) had fewer headache days initially, prophylaxis with flunarizine, and higher quality of life. In a study in China, a diagnosis of migraine versus tension headache and low education were associated with a higher relapse rate after withdrawal within 1 year [95]. Carlsen et al. demonstrated in an open-label study that complete medication withdrawal had a lower likelihood of relapse than treatment that allowed the use of medications for the treatment of migraine attacks on up to 2 days per week [96]. A study on long-term predictors of remission in a prospective study of 240 MOH identified lower number of headache days per month before 1-year follow-up and initial efficient medication withdrawal (> 50% symptom reduction or no overuse at symptom recurrence) as predictors [97].

However, there are no prospective controlled studies with intensive treatment of patients within the first year for sustained prevention of relapse. The question of whether prophylactic drug therapy initiated concurrently with a drug pause or withdrawal prevents relapse also cannot be answered unequivocally. However, it has been shown that the combination of a medication pause with an intensive outpatient, day-care or inpatient psychoeducational treatment program with motivational elements is highly effective and cost-saving [98].

## Data Availability

Does not apply.

## Abbreviations

CGRP: Calcitonin gene related peptide
COPD: Chronic onstructive pulmonary disease
DGN: German Society of Neurology
DMKG: German Migraine and Headache Society
EMG: Electromyography
ICDH: International Classification of Headache Disorders
IHS: International Headache Society
NRS: Numerical Rating Scale
NSAID: Non-steroidal antiinflammtory drug
MO: Medication overuse
MOH: Medication overuse headache
TIA: Transient ischemic attack
